# Self-construal priming selectively modulates the scope of visual attention

**DOI:** 10.3389/fpsyg.2015.01508

**Published:** 2015-09-30

**Authors:** Zhuozhuo Liu, Menxue Cheng, Kaiping Peng, Dan Zhang

**Affiliations:** Department of Psychology, School of Social Sciences, Tsinghua UniversityBeijing, China

**Keywords:** self-construal, priming, spatial-based attention, feature-based attention, culture

## Abstract

Self-concept is one of the major factors to explain the cultural differences between East Asians and Westerners. In the field of visual attention, most studies have focused on the modulation of visual spatial-based attention, whereas possible influences of culture or self-concept on other types of visual attention remain largely unexplored. The present study investigated the possible modulation of visual feature-based attention by self-concept, using a within-group self-construal priming design. The experiment paradigm employed visual stimuli consisted of two intermixing random dot clouds presented in the focal visual field with red and green colors. After primed with an interdependent, independent, or neutral self-construal, the participants were instructed to attend to one of the focally presented dot cloud and respond to occasional luminance decrement events of the attended dot cloud. The detection of the focal events was found to be significantly faster when exogenously cued by a peripheral dot cloud of either the same or different colors as the attended focal dot cloud (congruent/incongruent), compared to the uncued condition. More importantly, the self-construal priming took effect only on the reaction time (RT) differences between the congruent and incongruent cued conditions: the participants responded much slower to incongruent cued events than congruent cued events under interdependent self-construal priming, while the RT difference was significantly smaller under independent self-construal priming. A closer look on the results suggests that the attention scope is selectively modulated by self-construal priming, and the modulation is mainly reflected by varying the degree of suppression on the processing of the incongruent contextual stimuli that do not share visual features with the focal object. Our findings provide new evidences that could possibly extend the current understanding on the cultural influence on visual attention.

## Introduction

A growing body of evidence has suggested that culture influences human perceptual processes (Morris and Peng, 1994; Nisbett and Miyamoto, 2005; Han and Northoff, 2008; Han and Ma, 2014). While the field of cross-cultural psychology covers diverse research topics for a variety of cultures, the cultural difference between East Asians and Westerners is probably one of the most studied topics in this field. It is now well accepted that Westerners are more likely to attend to the focal object independently of the context (i.e., analytic perception), whereas Eastern Asians tend to pay more attention to the relationship between the focal object and its contextual background (i.e., holistic perception; Kitayama et al., 2003; Nisbett and Miyamoto, 2005). This cultural effect has been observed in a variety of experiment paradigms, including rod-and-frame test (Ji et al., 2000), frame line test (Kitayama et al., 2003; Hedden et al., 2008), Navon test (Kühnen and Oyserman, 2002; McKone et al., 2010), change blindness test (Masuda and Nisbett, 2006), flanker test (Lin and Han, 2009), etc. Compared with Westerners, East Asians’ performances of the focal behavioral tasks were in general more likely to be influenced by the context surrounding the visual attention focus.

Self-concept has been regarded as one of the major factors to explain the cognitive differences between East Asians and Westerners. While East Asians are associated with interdependent selves emphasizing relationships and fitting in with others, Westerners are associated with independent selves focusing on the appreciation of one’s difference from others and the importance of self assertion (Markus and Kitayama, 1991). More importantly, it has been suggested that both the interdependent and the independent self-construals coexist in all cultures; people can be temporarily primed to one of the self-construals (Gardner et al., 1999). In the field of cultural influences on attention, researchers have found that both East Asians and Westerners were subject to self-construal priming, with interdependent self-construal leading to a more context-dependent attention mode, or a broader scope of attention (Hsu, 1971; Kühnen and Oyserman, 2002; Cha et al., 2005; Lin and Han, 2009). In contrast to the between-group cultural studies, most of these priming studies were carried out in a within-group manner, i.e., the same group of participants primed to different self-construals within the same experiment. Hereby, the observed behavioral differences under different priming conditions support the casual consequences of self-knowledge on attention. These results suggest that self-knowledge modulates not only the high-level social information processing, but also perceptual information processing at a basic cognitive level.

To date, most of the findings on cultural differences of attention belong to the category of visual spatial-based attention, as the focal object and the context background were usually with distinct spatial properties. The visual feature-based attention, however, remains largely unexplored. In contrast to the ‘spotlight’ model of visual spatial-based attention, studies on visual feature-based attention described another important mechanism of visual attention allocation that focuses on the representation of visual image components throughout the visual field that are related to a particular feature, such as color, motion direction, shape, etc. (Rossi and Paradiso, 1995; Treue and Maunsell, 1999; Saenz et al., 2003; Maunsell and Treue, 2006). One of the most distinguished differences between spatial-based attention and feature-based attention is that feature-based attention can operate globally on stimuli outside the attended spatial location, leading to facilitated processing of the feature-sharing stimuli compared to all other stimuli. Moreover, neuroimaging studies have shown that feature-based attention results in both enhanced cortical responses to the attended visual features and suppressed responses to the unattended features (Saenz et al., 2002; Müller et al., 2006; Zhang and Luck, 2009; Andersen and Müller, 2010), with similar response timing as spatial-based attention (Zhang and Luck, 2009). Therefore, it has been proposed that feature-based attention operates independently from spatial-based attention.

Although feature-based attention has not been directly tested in cultural difference studies, a number of previous cultural or self-concept related studies have employed pairs of focal and contextual stimuli sharing similar visual features such as colors or shapes (Kühnen and Oyserman, 2002; Kitayama et al., 2003; Hedden et al., 2008; Lewis et al., 2008; McKone et al., 2010). Although their findings were explained in the direction of spatial-based attention, i.e., a broader scope of visual spatial-based attention by East Asian culture or interdependent self-construal priming, these results are, to some extent, consistent with the feature-based attention mechanisms as well: the influence of the focal attention task by the contextual background may be explained by the allocation of attention due to feature sharing between focal and contextual stimuli. Culture, or self-construal, modulates the scope of visual attention, which in turn determines the degree of influence by the feature-sharing stimuli from the contextual background. However, it remains to be elucidated whether the attention scope is unconditionally modulated, or selective only for contextual stimuli that share features with the focal object. The answer to this question cannot be simply derived from the previous studies, as most of these studies did not introduce competing visual stimuli with distinct visual features in the contextual background. Therefore, it is necessary to design new experiment paradigms that directly address this question from a feature-based attention perspective, which may further extend our current understanding about the cultural modulation of the attention scope.

In the present study, we employed a revised version of one classical visual feature-based attention paradigm (Zhang and Luck, 2009) to investigate the influence of visual feature-based attention by self-construal priming. In our paradigm, the participants were presented with intermixing red and green random dots in their visual attention center as the focal stimuli. The participants’ task was to detect and respond to occasional luminance decrements in the attended color. The luminance decrements might be preceded (50% chance) by the presentation of a short-lasting random dot cloud presented in the visual periphery as the contextual background stimuli, with either the same or different colors compared to the to-be-attended color (i.e., congruent or incongruent cues). The peripherally presented dots are supposed to serve as an exogenous cue that facilitates the detection of the focal luminance decrements, leading to faster reaction times (RTs; Posner, 1980; Yantis and Jonides, 1990) for the cued focal task than for the uncued focal task, namely the exogenous cuing effect. The modulation of the visual spatial-based attention by self-concept is expected to result in a significant difference of the exogenous cuing effect under the interdependent and the independent self-construal priming conditions. Specifically, the interdependent self-construal (i.e., representative of East Asians) is hypothesized to be associated with broadening of the attention scope, leading to a better perception of the exogenous cue and consequently a stronger exogenous behavioral effect. More importantly, we are interested in the possible influences of visual feature-based attention on the exogenous cuing effect, i.e., whether the congruent and incongruent cues could exhibit distinct behavioral performances under different self-construal priming conditions. Hereby, no significant interaction between the cuing conditions (congruent vs. incongruent) and the self-construal priming conditions would suggest that our attention is modulated by self-construal in a spatial manner only. However, an interaction may indicate that self-construal selectively modulates our attention by emphasizing the visual stimuli sharing the same color feature as the to-be-attended focal visual stimuli.

The present study was conducted using a within-group design: the participants were primed to different self-construals prior to taking the experiment, using a pronoun circling manipulation. Such a within-group design enables us to explore the casual relationship of self-construal and visual attention (Gardner et al., 1999; Kühnen and Oyserman, 2002; Sui and Han, 2007; Lin and Han, 2009). The aim of this study is two-folded. Firstly, we examined whether the modulation of the spatial scope of visual attention by self-construal priming could be replicated in this new experiment paradigm. Secondly, we further explored the influence of self-construal on feature-based attention, i.e., whether the attention scope is selectively modulated.

## Materials and Methods

### Subjects

Thirty-four Chinese students (21 males and 13 females) in Tsinghua University between 18 and 25 years-old participated in the study (mean 21 years). All had normal visual acuity and normal color vision. All were right-handed and were naïve to the purpose of the study. They gave their informed consent. The purpose of the study was revealed to the participants after the completion of the experiment. The study was conducted in accordance with the Declaration of Helsinki, 2004 and approved by the local ethics committee of Tsinghua University.

### Materials

**FIGURE 1:**
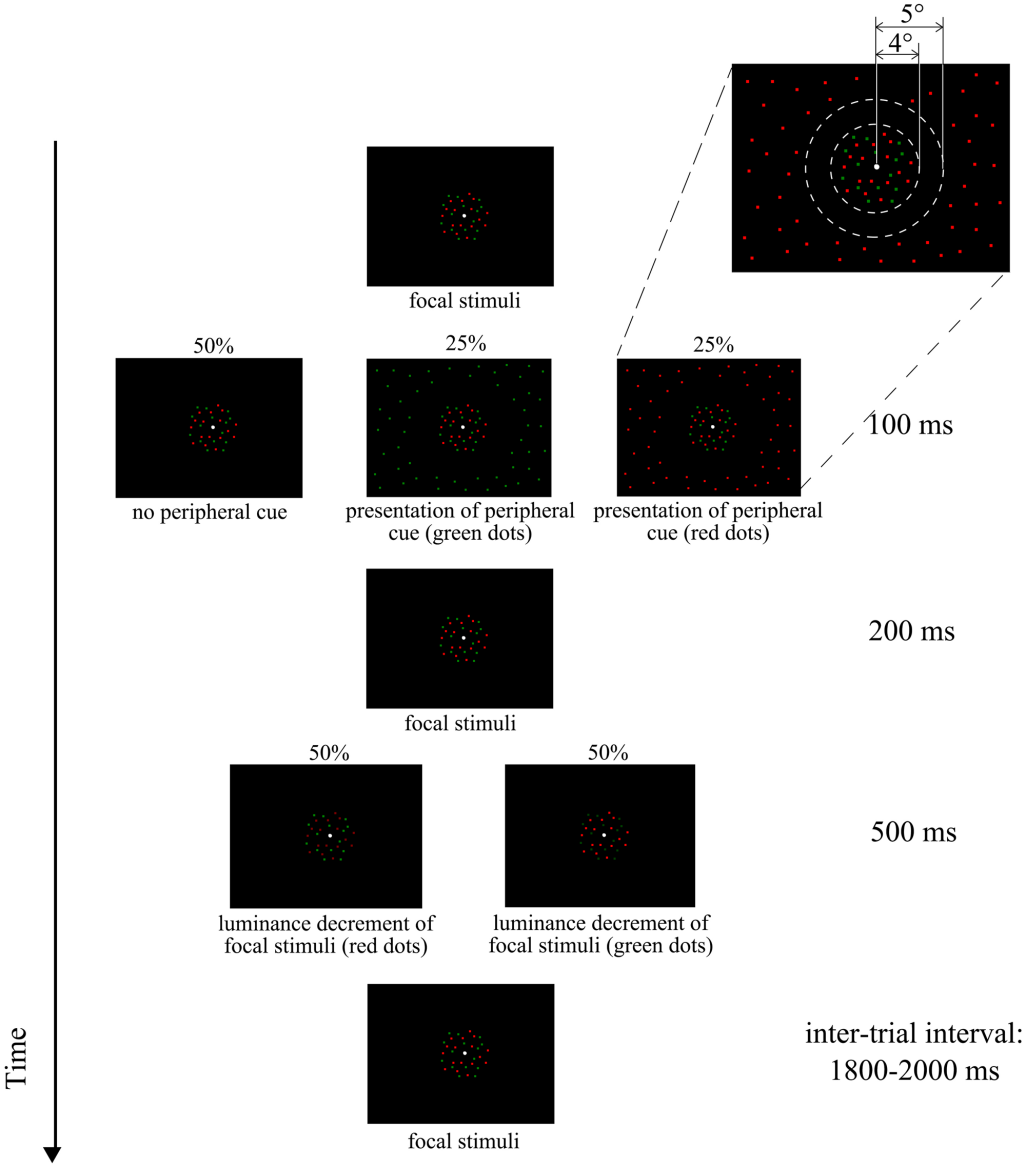
**Illustration of the stimulus and the timing of one trial**.

An illustration of the stimuli and the experiment timing of one trial are provided in **Figure 1**. The stimuli were presented on a 23-inch computer monitor (DELL, USA) with a resolution of 1920 × 1080 pixels, and 60 Hz refresh rate. The viewing distance was 50 cm. One white dot subtending a visual angle of 0.15°, was always presented at the center of the screen for fixation. Task-relevant stimuli were two dot sets (50 dots each) of different colors (red and green) and equal brightness randomly distributed in an annular area within 4.0° from the fixation dot (i.e., the focal region). Each dot subtended 0.15° of visual angle. To minimize the possibility of sustained attention on a single dot, half of the dots disappeared and reappeared in another random location every 100 ms. These random dots were continuously presented throughout the experimental blocks with no blank screen between consecutive trials (see *Procedure* for the definition of blocks).

Occasional luminance decrements occurred every 1800–2000 ms during the presentation of these random dots. The luminance decrements of the red dots were achieved by changing the red color from RGB (255, 0, 0) to RGB (120, 0, 0). The green dots were manipulated in a similar way, by changing the color from RGB (0, 128, 0) to RGB (0, 60, 0). These RGB values were determined in a pilot study with a group of 10 participants, for subjective equal brightness for the original colors as well as a similar level of luminance decrements for both red and green colors. Each luminance decrement consisted of the color change of all dots of a single color in the focal region, lasting for 500 ms.

Preceding the luminance decrements, there was a 50% chance of the presentation of another random dot cloud in the peripheral visual region, in an annular area from 5.0° away from the fixation dot, to the border of the screen (∼26° horizontally and 15° vertically). Each peripheral dot cloud consisted of 900 dots of a single color (either red or green). The dots were the same size as those in the focal region. The presentation duration was 100 ms and the interval between the presentation of the peripheral dot cloud and the focal luminance decrement was 200 ms.

The peripheral dot cloud served as an exogenous cue that was expected to affect the detection of the luminance decrements in the focal region of the attended color. In total, there were six different types of trials, depending on the peripheral-focal stimulus combinations. The trials were categorized by the factors of color of focal luminance decrement (red/green, 50% probability for each color) × peripheral cue type (50% chance with no cue, 25% chance with the red cue, and 25% chance with the green cue). The purpose of having 50% chance of no cue was to prevent the participants from having the impression of peripheral-focal stimulus cooccurance.

Manipulation of the self-construal level was done by employing a pronoun circling task (Gardner et al., 1999; Kühnen and Oyserman, 2002). Two Chinese travel stories were used in the priming procedure. Each story had three versions: one with interdependent pronouns (e.g., we, ours), one with independent pronouns (e.g., I, mine), and the third with no pronouns but only some neutral words (e.g., there is a mountain in Xizang). The participants were asked to read the stories and circle specific nouns in it (e.g., circle “we” and “ours” for interdependent priming, circle “I” and “mine” for independent priming and circle “mountain” and “river” for neutral priming). In each priming condition, the two stories of the corresponding version were used. The priming materials used in the present study have been applied previously and have been proved to be effective for Chinese university student cohorts (Sui and Han, 2007; Lin and Han, 2009).

### Procedure

The experiment consisted of a training block and three formal blocks. The training block consisted of one 56-trial session to make the participant familiarize with the task. As shown in **Figure 1**, each trial consisted of the presentation of one luminance decrement of either one of the focal dot clouds, preceded by an exogenous peripheral cue (50% chance). The 56-trial session included a random and continuous presentation of the following trials: seven trials of red cue + red decrement, seven trials of red cue + green decrement, seven trials of green cue + red decrement, seven trials of green cue + green decrement, 14 trials of no cue + red decrement, and 14 no cue + green decrement.

Each formal block was preceded by one specific priming procedure: the participants carried out the pronoun circling task on the two travel stories of the corresponding version. After the priming task, the participants completed one experimental block, consisting of 112 trials that were divided into two sessions. Each session consisted of 56 trials with the same trial categorical distribution as the training session. The participants were required to press the ‘J’ key on the computer keyboard as fast as possible after the detection of the luminance decrement of the to-be-attended color and ignore the luminance decrement of the other color. The inter-session intervals lasted usually less than 1 min (upper limit: 2 min) and the duration was controlled by the participants. After the completion of one block, the participants had a 5-min break to avoid the left-over priming effect of the last block, and then started to take the next priming task and another experimental block. The participants received no feedback about their behavioral performance during the experiment. The order of the priming tasks was randomized and counterbalanced across the participants (all six possible priming orders were employed; four out of the six orders were used six times and the remaining two orders were used five times).

The to-be-attended color was instructed at the beginning of the experiment and was fixed for each participant. The to-be-attended color was counterbalanced across the participants with the consideration of the priming orders (equal numbers of participants within each priming order sub-group were assigned to the two to-be-attended colors; for the two sub-groups with five participants, two/three and three/two participants were instructed to attend red/green, respectively). Each session lasted for approximately 4 min. The whole experiment lasted for about 50 min. Presentation of the stimuli was programmed in Matlab 7.7.0 (The Mathworks, USA) using the Psychophysics Toolbox 3.0 extensions (Brainard, 1997; Pelli, 1997).

### Data analysis

Responses occurring between 100 and 1000 ms after the onset of the luminance decrements were regarded as correct. Among the above-defined six trial types, only three of them needed to be responded, according to the to-be-attended color per participant. The RTs of these trials were categorized into three types according to the exogenous cues: no-cue, con-cue (i.e., the cue color was congruent with the color of the focal luminance decrement event), and incon-cue (i.e., incongruent colors between the cue and the focal event). These RTs could be labeled according to their priming conditions as well: interdependent-priming (inter), independent priming (inde), and neutral priming (neutral). Only RTs from the correctly responded trials were included for further statistical analysis. The false alarm rates were not analyzed, as the number of the erroneous responses to the luminance decrements of the to-be-ignored color was in most cases less than three per session.

To directly address our two research questions, two planned ANOVAs were performed. The spatial-based attention effect by self-construal was investigated by performing ANOVA with the repeated measurement factors Cue Presence (2: no-cue/con-cue + incon-cue) and Priming (3: inter/inde/neutral). The influence of self-construal on feature-based attention was explored by performing ANOVA with the repeated measurement factors Cue Congruency (2: con-cue/incon-cue) and Priming (3: inter/inde/neutral). As the two ANOVAs were conducted on the same dataset, the Bonferroni correction for multiple comparisons was performed the corrected *p*-values were reported (the original *p*-values were corrected by multiplying the number of comparisons and then compared to the statistical threshold, i.e., 0.05; for these two planned ANOVAs, *p*-values were multiplied by 2). RTs were also subjected to analyses of variance (ANOVA) with the repeated measurement factors Cue Condition (3: non-cue/con-cue/incon-cue) and Priming (3: inter/inde/neutral) to get an overall description of the results.

The Statistical Package for Social Sciences (SPSS 20.0, IBM, USA) was used for data analysis. For all repeated measures analyses, Greenhouse–Geisser adjustments were performed when necessary, and the adjusted *p* values were reported.

## Results

The behavioral performances are summarized in **Table 1**. The participants detected the luminance decrement events with an average accuracy of 92.0 ± 1.9% across all conditions. The accuracies did not differ significantly between conditions.

**Table 1:** Mean reaction times (RTs) (ms) and response accuracy (%) (SEM).

		Neutral	Interdependent	Independent
RTs	No cue	525.0 (9.6)	529.1 (10.0)	520.3 (8.8)
	Congruent Cue	446.5 (8.5)	447.3 (9.2)	444.7 (8.4)
	Incongruent Cue	456.9 (8.6)	463.4 (10.3)	448.8 (9.0)
Accuracy	No cue	89.0 (2.2)	89.1 (2.4)	90.2 (2.3)
	Congruent Cue	94.0 (1.5)	95.1 (1.2)	96.1 (1.1)
	Incongruent Cue	93.8 (1.6)	93.7 (1.7)	94.7 (1.3)


**FIGURE 2:**
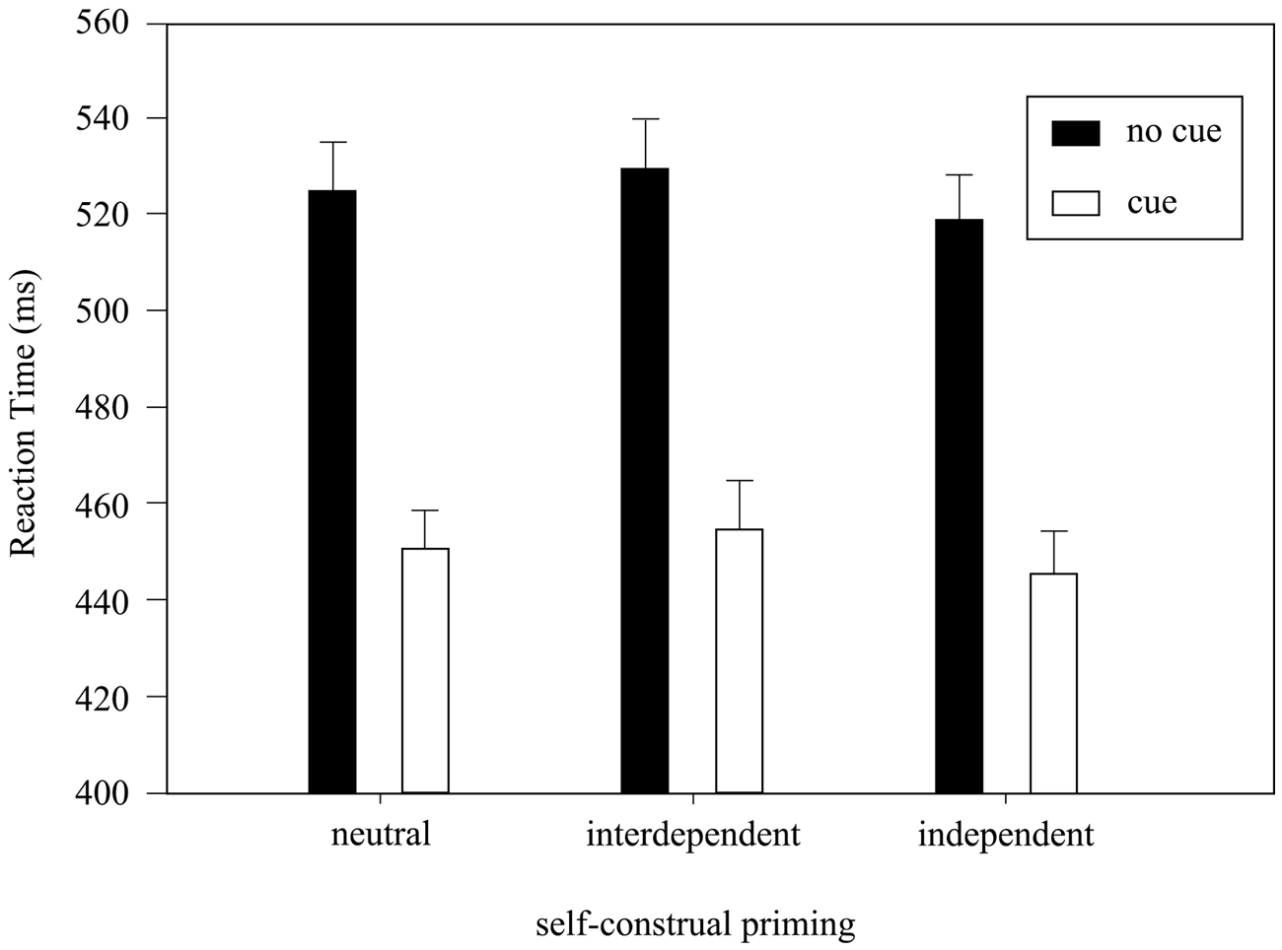
**The mean reaction times (RTs) by Cue Presence and Priming.** Error bar indicates standard error of the mean (SEM).

Analyses of variance analysis on RTs with factors Cue Presence and Priming revealed a significant main effect of Cue Presence [*F*(1,33) = 229.7, *p* < 0.001, $\eta_{p}^{2}$ = 0.87]. **Figure 2** shows the mean RTs for Cue Presence in the three priming conditions. The result showed a strong facilitation of the response speed by the presence of the exogenous cue [532.1 ± 9.6 ms (no-cue) vs. 457.2 ± 8.4 ms (con-cue + incon-cue)]. There were no other significant main effects or interactions.

**FIGURE 3:**
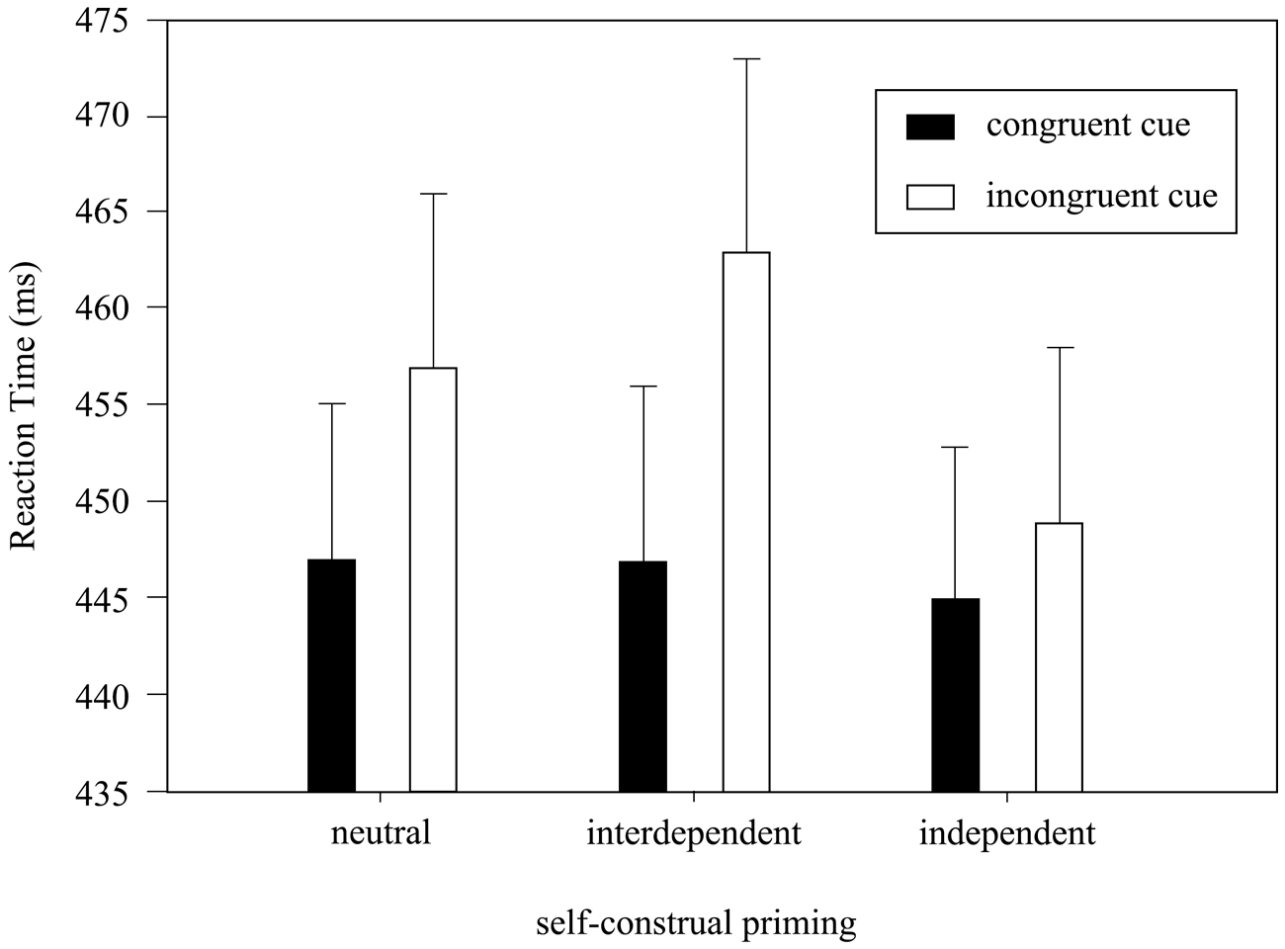
**The mean RTs by Cue Congruency and Priming.** Error bar indicates SEM.

Analyses of variance analysis on RTs with factors Cue Congruency and Priming revealed both a significant main effect of Cue Congruency [*F*(1,33) = 16.1, *p* < 0.001, $\eta_{p}^{2}$ = 0.33] and a significant interaction [*F*(2,66) = 4.0, *p* < 0.05, $\eta_{p}^{2}$ = 0.11]. **Figure 3** shows the mean RTs for Cue Congruency in the three priming conditions. RTs were in general lower when the participants responded to focal events preceded by a congruent cue, compared to an incongruent cue [452.8 ± 8.7 ms (con-cue) vs. 461.4 ± 8.4 ms (incon-cue)]. Moreover, the RT differences between the trials with congruent and incongruent cues were larger under the Interdependent Priming condition than those under the Independent Priming condition [inter: ΔRT = RT(incon-cue) – RT(con-cue) = 15.2 ± 2.1 ms, inde: Δ*RT* = 4.5 ± 1.6 ms, *t*(33) = 2.8, *p* < 0.01, *d* = 0.48]. The RT difference under the Neutral Priming condition was 10.4 ± 3.2 ms, which was in between the other two priming conditions.

The overall ANOVA on RTs with factors Cue Condition and Priming showed a significant main effect of Cue Condition [*F*(2,66) = 204.1, *p* < 0.001, $\eta_{p}^{2}$ = 0.86] and no other significant effects [main effect of Priming: *F*(2,66) = 1.1, *p* = 0.36, $\eta_{p}^{2}$ = 0.03; interaction: *F*(4,132) = 1.8, *p* = 0.15, $\eta_{p}^{2}$ = 0.05]. Although the overall interaction did not reach a significant level, we regard the two planned ANOVAs as valid for the following considerations. (1) The statistical power of our planned ANOVAs might be underestimated in the overall ANOVA. (2) The power of the overall ANOVA might also be reduced due to the unbalanced trial numbers in the three cue conditions: the number of trials in the no-cue condition is twice as many as the number of trials in the con-cue and incon-cue conditions, respectively. In contrast, the number of trials for obtaining the average RTs was balanced in both of our planned ANOVA, which might lead to increased statistical sensitivity.

## Discussion

The present study investigated the modulation of both visual spatial-based and feature-based attention by self-construal, using a focal-peripheral random dot paradigm. The peripherally presented cue indeed triggered exogenous attention, leading to faster RTs for the follow-up focal detection task, compared with the uncued condition. However, the facilitation of the RTs by the presence of the peripheral cue did not differ among self-construal priming conditions. In contrast, RTs to exogenous cues that were congruent or incongruent with colors of the to-be-attended color of the focal task, showed a significant difference by self-construal priming: interdependent priming was associated with the largest RT differences between congruent and incongruent cues.

While most studies on exogenous attention have employed exogenous cues from different spatial locations and compared the RT differences between responses to targets from cued and uncued locations (Posner, 1980; Yantis and Jonides, 1990; Chica et al., 2011), here we used a simplified version without the spatial information. The peripherally presented dot cloud was expected to exogenously capture the participants’ attention to the computer screen as a whole, therefore facilitating the response to the focal stimulus as well. Indeed, we observed a significant RT difference: the cued focal luminance decrements were responded about 70 ms faster than the uncued ones. Our results were of the same magnitude as those reported in previous studies (see Chica et al., 2013, for a review), illustrating the effectiveness of our experimental manipulation.

The cued vs. uncued RT differences, however, did not differ significantly for the three self-construal priming conditions. Such an observation cannot be simply explained by the state-of-art understanding of the cultural influences on visual attention (Nisbett and Miyamoto, 2005; Lin and Han, 2009; McKone et al., 2010): if the interdependent priming indeed broadened the scope of visual attention, the exogenous cue would have a better capture of the participants’ attention, leading to faster cued RTs under the interdependent priming condition, compared to the other two conditions. As no slight trend toward this direction was detected (**Table 1**; **Figure 2**), our results call for an extension of the present spatial attention based hypothesis.

Our analyses of the RTs by Cue Congruency and Priming provide an explanation for the above-mentioned inconsistency, possibly from a feature-based attention perspective. The RTs under the congruent cued condition were significantly faster than those under the incongruent cued condition, demonstrating an effective manipulation of visual feature-based attention (Saenz et al., 2003; Zhang and Luck, 2009). More importantly, the interaction between Cue Congruency and Priming revealed a possible influence of self-construal priming on visual feature-based attention. The RT difference between the congruent and incongruent cued conditions was largest (∼15.2 ms) when the participants were primed to the interdependent self-construal. Such an effect could be explained in a similar way as the previous attention scope hypothesis but with feature-based attention incorporated. The interdependent self-construal (i.e., representative of the East Asians) might result in a broadening of the attention scope together with a biased information processing in favor of the visual stimuli that sharing the same feature (e.g., color etc.) as the focally attended stimulus. As the modulation of RTs by priming under the congruent cued conditions showed a maximal RT difference of less than 5 ms but the counterpart under the incongruent cued conditions varied as much as ∼15 ms, our results demonstrate asymmetric enhancement and suppression of visual feature-based attention that mainly showed a suppressed processing of the unattended visual features, rather than an enhanced processing of the attended visual features. Specifically, the broadening of the attention scope by interdependent self-construal might lead to a stronger suppression of the incongruent cues, as a larger proportion of the peripheral stimuli was likely to be within the attention scope. The consequently impaired perception of the incongruent cues might render their exogenous cuing effect less prominent, resulting in slower RTs.

The seemingly contradictable finding in the present study, however, does not actually violate the previous findings from both self-concept based and cultural studies (Masuda and Nisbett, 2001; Hannover et al., 2005; Gutchess et al., 2006; Miyamoto et al., 2006; Boduroglu et al., 2009; Lin and Han, 2009; McKone et al., 2010; Springer et al., 2012). To our knowledge, all of the previous experiments have only divided the visual stimuli into focal and context (i.e., peripheral), without further differentiation of the context. In our study, we deliberately introduced focal-context congruency and we did find a significant modulation of the RT differences by priming. Our finding suggests that the previous understanding is an oversimplified version of the self-construal (and possibly cultural) modulation on visual attention.

It is also worth noting that the RT differences between congruent and incongruent cued conditions under the neutral priming condition lay just in between the interdependent and independent priming. Although Chinese participants were conventionally expected to have a neutral baseline more toward the interdependent self-construal, the recruited participants in the present study may be less typical as these university students had sufficient exposures to Western cultures and most of them have been abroad for either long-term or short-term stays. Hereby, their “bicultural” background is preferred for our self-construal priming, as they could be more easily primed to different self-construals than people with no knowledge about Western cultures (Hong et al., 2000; Kitayama et al., 2003; Peng and Knowles, 2003). Nevertheless, future studies using different cultural cohorts together with self-construal priming may help us extend the present findings.

In addition, the experimental paradigm of the present study can be employed for further neurophysiological studies on the cultural modulation on visual feature-based attention. In contrast to the studies using complex pictures or photographs with possible cultural semantics (e.g., Masuda and Nisbett, 2001; Gutchess et al., 2006; Miyamoto et al., 2006), the visual stimuli used in the present study can be considered to be free of cultural semantic content (i.e., only random dots of red and green colors). Simple and abstract visual stimuli have also been adopted in previous studies (e.g., Ji et al., 2000; Kitayama et al., 2003; Boduroglu et al., 2009), and findings in these studies are believed to provide evidences for culture differences at a basic cognitive level. Therefore, the present paradigm is can be potentially applicable for investigating the underlying neural mechanisms of the cultural differences of visual feature-based attention, by incorporating neuroimaging techniques such as EEG, fMRI etc. One possible direction worth investigating could be the observed asymmetric enhancement and suppression effect at the behavioral level in the present study: would the asymmetric bias in visual feature processing also reflected at the neural level, or was it just because of the limitation of the behavioral study?

In sum, our study provides behavioral evidences that extend our current understanding of the self-construal effect on visual attention. Our findings suggest that the attention scope is selectively modulated by self-construal priming, and the modulation is mainly reflected by varying the degree of suppression on the processing of the incongruent contextual stimuli that do not share visual features with the focal object. Together with the within-group design and the priming-based manipulation of the self-concept, our findings argue for a possible influence on feature-based attention by self-construal.

## Conflict of Interest Statement

The authors declare that the research was conducted in the absence of any commercial or financial relationships that could be construed as a potential conflict of interest.
